# Early Real-World Outcomes of Switching to 8 mg Aflibercept for Neovascular Age-Related Macular Degeneration in the United Kingdom

**DOI:** 10.3390/life15060903

**Published:** 2025-06-02

**Authors:** Muiz Musadiq, Mohammed Musadiq, Fozia Latif, Benjamin Ng, Matthew Azzopardi, Noa Gilead, Andrew Needham, Yu Jeat Chong

**Affiliations:** 1 School of Medicine, University of Liverpool, Liverpool L69 3GE, UK; muizmusadiq@gmail.com; 2 Eyecare Medical, Macclesfield SK11 7JW, UK; mohammed.musadiq@uhnm.nhs.uk (M.M.); fozilat@hotmail.co.uk (F.L.); andrew.needham@eyecaremedical.co.uk (A.N.); 3 Department of Ophthalmology, University Hospital of North Midlands, Stoke-on-Trent ST4 6QG, UK; 4 Nuffield Department of Clinical Neurosciences, University of Oxford, Oxford OX3 9DU, UK; benjamin.ng.wj@gmail.com; 5 Moorfields Eye Hospital, City Road, London ECV1 2PD, UK; matthew.azzopardi.14@um.edu.mt; 6 Ophthalmology Department, Kaplan Medical Centre, Rehovot 7661041, Israel; noagilead@gmail.com; 7 Birmingham and Midland Eye Centre, Birmingham B18 7QH, UK

**Keywords:** aflibercept 8 mg, treatment switching, treatment refractory neovascular age-related macular degeneration

## Abstract

(1) Aim: To evaluate early real-world outcomes of switching to aflibercept 8 mg in eyes with neovascular age-related macular degeneration (nAMD) in the United Kingdom. (2) Methods: This retrospective, observational study included 59 eyes from 50 patients with treatment-refractory nAMD previously treated with multiple anti-vascular endothelial growth factor (anti-VEGF) agents. Eyes were switched to aflibercept 8 mg without loading doses and treated using a treat-and-extend regimen. Functional, anatomical, and safety outcomes were evaluated over a mean (SD) follow-up of 33.5 (10.4) weeks. (3) Results: The mean (SD) age was 80.2 (6.3) years, and 28 (56.0%) of 50 patients were male. At baseline, the mean (SD) best corrected visual acuity (BCVA) was 66.0 (14.4) letters, with 33 (55.9%) eyes achieving ≥70 letters. The mean (SD) baseline central subfield thickness (CST) was 367.2 (100.7) µm. Prior to switching to aflibercept 8 mg, the mean (SD) number of injections for each eye was 26.9 (19.0), with the most recent mean (SD) treatment interval of 7.7 (1.7) weeks. Switching to aflibercept 8 mg resulted in extension of the mean (SD) injection interval from 7.7 (1.7) weeks to 8.7 (2.2) weeks (*p* < 0.01). BCVA and CST remained stable, with a significant reduction in pigment epithelial detachment (PED) height (232.5 µm to 211.6 µm, *p* < 0.01). No serious ocular adverse events or intraocular pressure (IOP) elevations requiring treatment were reported. (4) Conclusion: Aflibercept 8 mg demonstrated early treatment durability, anatomical benefit, and a favourable short-term safety profile in eyes with treatment-refractory nAMD. Further prospective studies are warranted.

## 1. Introduction

Neovascular age-related macular degeneration (nAMD) is a leading cause of vision loss in older adults in developed countries [1]. Vision loss occurs due to macular neovascularisation, where abnormal blood vessels proliferate and leak—leading to haemorrhages, accumulation of subretinal (SRF) and intraretinal (IRF) fluid, and ultimately fibrosis [2]. The current pharmacological standard of care for nAMD involves intravitreal injections of anti-vascular endothelial growth factor (anti-VEGF) agents [3].

Within the United Kingdom’s (UK) National Health Service (NHS), several anti-VEGF agents have been used, including bevacizumab, ranibizumab, aflibercept, brolucizumab, and faricimab [4,5]. In February 2024, aflibercept 8 mg, a higher-dose formulation of aflibercept, was approved for use in the UK [6]. It delivers a four-fold increase in molar concentration in comparison to aflibercept 2 mg and is designed to provide a more sustained VEGF suppression, with the goal of improving durability and anatomical outcomes [7]. Its approval was supported by the PULSAR trial, which demonstrated non-inferiority in best corrected visual acuity (BCVA) gains at 48 weeks in treatment-naïve eyes with nAMD receiving aflibercept 8 mg every 12 or 16 weeks, compared to 8-weekly aflibercept 2 mg [8]. The trial also reported earlier fluid resolution and durable anatomical control through 96 weeks, with over 80% of patients maintaining ≥12-weekly treatment intervals [7,8].

While these outcomes are promising, they were observed in carefully selected, treatment-naïve eyes under fixed dosing protocols. In contrast, real-word practice, particularly within the NHS, has largely adopted the treat-and-extend (T&E) approach [9]. This regimen individualises injections based on disease activity, aiming to reduce treatment burden while maintaining disease control [10]. In cases of suboptimal treatment response, NHS guidelines recommend reviewing treatment and considering a switch to an alternative anti-VEGF agent [11]. Several studies have shown that switching agents in treatment-refractory eyes with persistent IRF/SRF can lead to anatomical improvements, although visual outcomes have been more variable [12,13,14].

Whether aflibercept 8 mg can deliver similar benefits in treatment-refractory eyes despite previous intensive anti-VEGF regimens remains unclear. To the best of our knowledge, real-world data on the use of aflibercept 8 mg in previously treated nAMD within the UK and NHS setting are currently limited. In this study, we aim to address this gap in the literature by evaluating the early real-world efficacy, durability, and safety of aflibercept 8 mg in eyes with previously treated nAMD.

## 2. Materials and Methods

This retrospective, real-world observational study was conducted at a single NHS provider clinical site in the UK (Eyecare Medical, Macclesfield). As a retrospective service evaluation at a single centre, it was reviewed and approved by the clinic’s Clinical Governance team and conducted in accordance with the principles of the Declaration of Helsinki. As such, formal ethics committee approval was not required. Patient confidentiality was maintained throughout, and all data were anonymized prior to analysis. Patient visits occurred between February 2024 and January 2025, with January 2025 serving as the data cut-off for this interim analysis.

Eligible eyes had nAMD, were previously treated with more than one anti-VEGF agents, and were subsequently switched to aflibercept 8 mg. There were no exclusion criteria. While no formal exclusion criteria were applied, eyes with media opacities that precluded adequate OCT imaging or with end-stage disease judged unsuitable for further anti-VEGF therapy were not considered for treatment switch in routine practice, and, therefore, not included in this cohort.

### 2.1. Data Collection

Baseline data included patient demographics (age, sex), lens status, BCVA, and anti-VEGF treatment history. Optical coherence tomography (OCT) parameters assessed were the presence of SRF, IRF, and/or pigment epithelial detachment (PED), central subfield thickness (CST), maximum central retinal thickness (Cmax), and PED height. Follow-up data included the number of injections, injection intervals, intraocular pressure (IOP) measurements, and both anatomical and functional outcomes.

### 2.2. Treatment Protocol

Patients who switched to aflibercept 8 mg continued treatment using an interval-matched approach based on their most recent injection interval prior to the switch. No loading doses were given.

A T&E regimen with a treat-to-dry strategy was used. OCT features including SRF, IRF, and PED were assessed qualitatively. If SRF or IRF persisted, the injection interval was maintained. In cases of worsening or new disease activity, such as increased SRF/IRF, new choroidal neovascular membrane (CNVM), new subretinal hyperreflective material (SHRM), or haemorrhage, the interval was shortened by two weeks. If fluid improved or reduced, the interval was extended by two weeks, up to a maximum of 12-weekly intervals. PED alone did not guide interval adjustment unless it was deemed to be worsening, typically indicated by an increase in PED height.

### 2.3. OCT Imaging

Macular OCT was performed using the Spectralis OCT system (Heidelberg Engineering, Heidelberg, Germany), with a scanning protocol consisting of 19 B-scans covering a 30 × 15-degree area, with each B-scan averaged from 9 individual scans. Retinal layer segmentation was reviewed by a senior consultant ophthalmologist and manually corrected when necessary. PED height was defined as the distance between Bruch’s membrane and the outer surface of the retinal pigment epithelium (RPE), measured using the Spectralis caliper function.

### 2.4. Statistical Analysis

Continuous variables are presented as mean and standard deviation (SD), while categorical variables are reported as frequency and percentage. Normality was assessed using the Shapiro–Wilk test. Paired comparisons were performed using the paired t-test for normally distributed data and the Wilcoxon signed-rank test for non-normally distributed data. McNemar’s test was used for paired categorical comparisons. Univariable binomial logistic regression was used to predict whether eyes were classified as having vision loss or no vision loss. All tests were two-sided, with statistical significance set at *p* < 0.05. Analyses were conducted using R statistical software version 4.3.3.

## 3. Results

### 3.1. Baseline Characteristics

In total, 59 eyes from 50 patients were included in the study. The mean (SD) age of the patients was 80.2 (6.3) years. A total of 28 (56.0%) of 50 patients were male, and all patients (100%) were of white ethnicity. Of the total, 28 (46.7%) eyes were pseudophakic.

The mean (SD) baseline BCVA letters was 66 (14.4) (Snellen equivalent 6/15), with 33 (55.9%) eyes recording ≥70 letters (Snellen equivalent 6/12). At the time of switch, the mean (SD) baseline CST was 367.2 (100.7) µm, Cmax was 487.3 (146.6) µm, and PED height was 232.5 (162.4) µm. The proportions of eyes with SRF, IRF, and PED were 66.1% (n = 39), 42.4% (n = 25), and 94.9% (n = 56), respectively. 53 (89.1%) eyes had either SRF or IRF.

Prior to switching to aflibercept 8 mg, the mean (SD) number of injections for each eye was 26.9 (19.0), with the most recent mean (SD) treatment interval of 7.7 (1.7) weeks. The majority of eyes were switched from faricimab (n = 35; 59.3%), while the rest were switched from aflibercept 2 mg (n = 22; 37.3%) and brolucizumab (n = 2; 3.4%).

A total of 76.3% (n = 45 eyes) were switched because of persistent disease activity. The other eyes were switched to either extend the treatment interval (n = 8 eyes; 13.6%), or because of new disease activity while under T&E (n = 6 eyes; 10.2%).

These baseline characteristics are summarised in Table 1.

### 3.2. Treatment Summary

Following the switch to aflibercept 8 mg, the mean (SD) number of injections per eye was 5.1 (1.4). The mean (SD) number of follow-up visits was 4.2 (1.6), while the mean (SD) follow-up duration was 33.5 (10.4) weeks.

**Figure 1:**
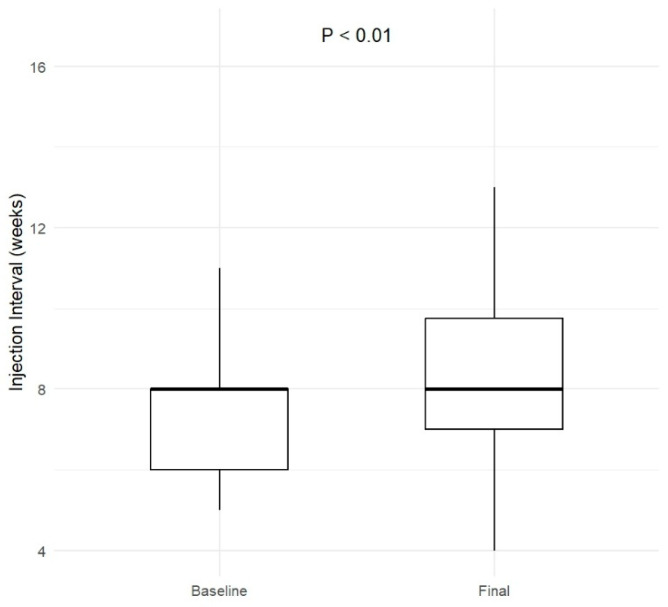
Injection interval at baseline and final visit. Box plots show the distribution of treatment intervals (in weeks) at both time points. A statistically significant increase in injection interval was observed at the final visit (*p* < 0.01).

**Figure 2:**
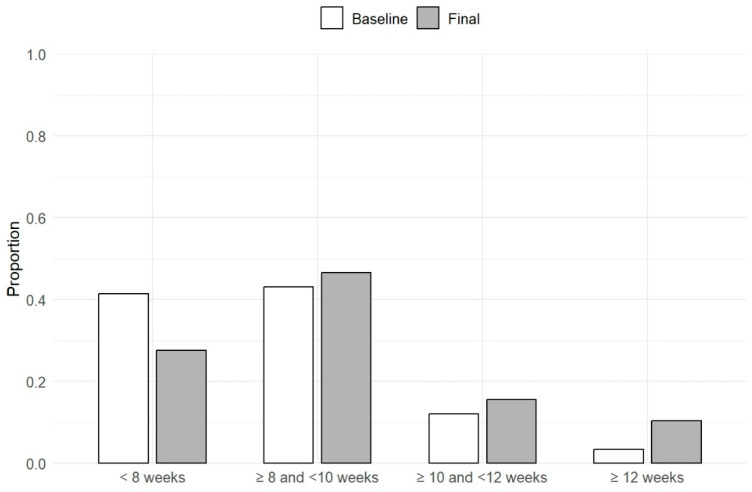
Distribution of eyes across injection interval categories at baseline and final visit. Bar heights represent the proportion of eyes receiving injections at intervals of <8 weeks, 8 to <10 weeks, 10 to <12 weeks, or ≥12 weeks at each time point.

The mean (SD) injection interval at the first follow-up visit was 8.4 (3.5) weeks. This increased to 8.7 (2.2) weeks at the final visit, which was significantly longer compared to baseline, *p* < 0.01. At the final follow-up, the majority of eyes (n = 27; 45.8%) were on an injection interval between ≥8 and <10 weeks. A total of 10 (16.9%) eyes were on an interval between ≥10 and <12 weeks, while 6 (10.2%) eyes were on an interval of ≥12 weeks. These results are summarised in Figure 1 and Figure 2.

### 3.3. Functional and Anatomical Outcomes

Following the first dose of aflibercept 8 mg, of the 53 (89.8%) eyes that had either SRF or IRF at baseline, 8 (15.1%) showed complete resolution of fluid and 24 (45.2%) demonstrated a decrease in SRF or IRF. Conversely, 19 eyes (41.3%) showed an increase in SRF or IRF following the first dose of aflibercept 8 mg.

**Figure 3:**
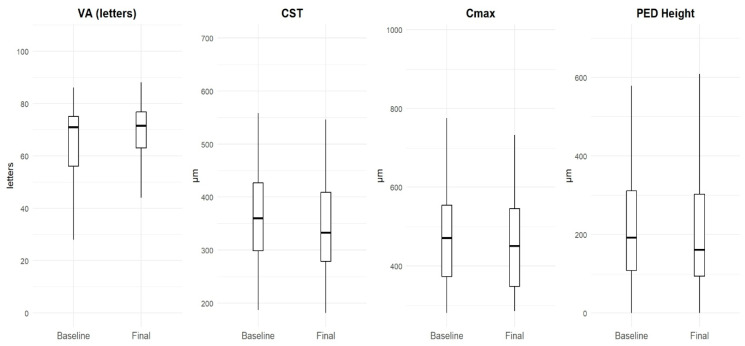
Changes in functional and anatomical markers from baseline to final visit. Box plots show distributions of visual acuity (VA, letters), central subfield thickness (CST, µm), maximum central retinal thickness (Cmax, µm), and pigment epithelial detachment height (PED, µm) at baseline and final assessments.

The mean (SD) CST, Cmax, and PED height at the final visit were 355.1 (107.7) µm, 471.8 (139.3) µm, and 211.6 (150.5) µm, respectively. The CST and Cmax were not significantly different from baseline (*p* = 0.21 and *p* = 0.28, respectively). There was, however, a statistically significant reduction in PED compared to baseline (*p* < 0.01). These results are summarized in Figure 3.

**Figure 4:**
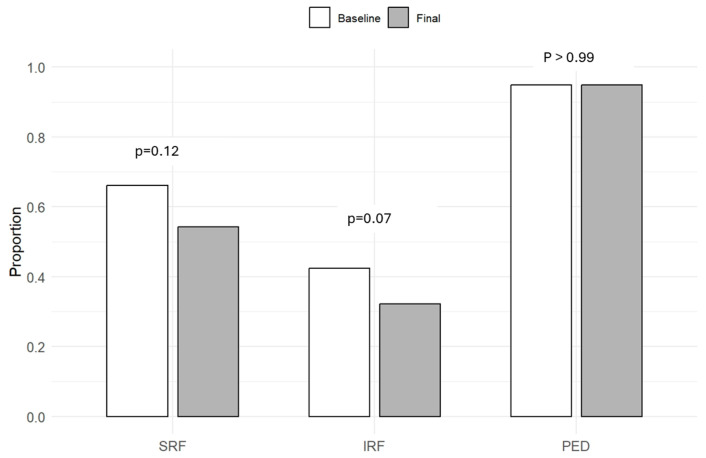
Bar graphs display the proportion of eyes with subretinal fluid (SRF), intraretinal fluid (IRF), and pigment epithelial detachment (PED) at baseline and at the final follow-up. *p*-values indicate comparisons between time points for each feature, none of which were statistically significant.

In terms of proportions of eye with SRF, IRF, or PED at final visit, none were different statistically compared to baseline (*p* > 0.05 for all): 32 eyes (54.2%) with SRF; 19 eyes (32.2%) with IRF; 56 eyes (94.9%) with PED. These results are shown in Figure 4.

### 3.4. Visual Acuity Outcomes

At the final visit, the mean (SD) BCVA was 68.2 (12.9) letters, and was not statistically significant from baseline (*p* = 0.86). The percentage of eyes with BCVA ≥ 70 letters was 49.2% (n = 29 eyes), less than baseline, although this was not statistically significant (*p* = 0.22).

**Figure 5:**
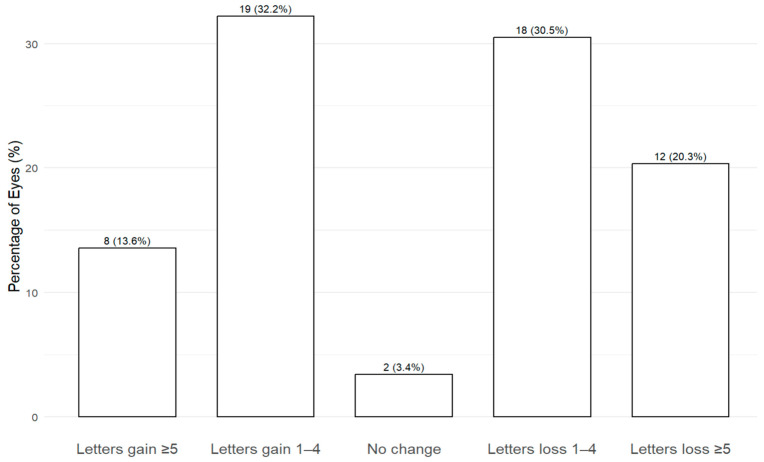
Distribution of visual acuity outcomes at final visit. Bars represent the percentage of eyes that experienced ≥5 or 1–4 letter gain, no change, or 1–4 or ≥5 letter loss compared to baseline. The number of eyes and corresponding percentages are shown above each bar.

In terms of letters gain, 19 eyes (32.2%) gained between 1 to 4 letters, and 8 eyes (13.6%) gained ≥5 letters. As for letters loss, 18 eyes (30.5%) lost between 1 to 4 letters, and 12 eyes (20.3%) lost ≥5 letters. 2 eyes (3.4%) had no change in BCVA. This is represented in Figure 5.

### 3.5. Predictors of Visual Acuity Loss

None of the predictor variables including age, gender, lens status, indication for switching, number of previous IVT injections, previous IVT interval, baseline BCVA, CST, Cmax, or PED height were significantly associated with BCVA loss (*p* > 0.05 for all). However, patients who received aflibercept 2 mg as their most recent intravitreal injection had significantly lower odds of BCVA loss compared to those treated with faricimab (odds ratio = 0.28, 95% confidence interval: 0.085 to 0.828, *p* = 0.03).

### 3.6. Safety

No eyes required IOP–lowering treatment at any visit, and none required emergency paracentesis. One patient with pre-existing cardiovascular risk factors experienced a documented stroke between their third and fourth intravitreal injections (with an injection interval of 15 weeks). As a result of the stroke, the patient’s follow-up was delayed, and the interval between their third and fourth injections was 15 weeks. This was not a protocol-driven extension. There were no other documented adverse events such as retinal vasculitis or endophthalmitis.

## 4. Discussion

We present outcomes from an early real-world retrospective observational cohort of eyes with treatment-refractory nAMD who were switched to aflibercept 8 mg and managed using a standardized T&E regimen. The majority of eyes were followed for over 6 months, providing early insights into the use of aflibercept 8 mg in routine NHS practice in the UK. This differs from the PULSAR trial, which evaluated aflibercept 8 mg in treatment-naïve eyes under fixed dosing schedules in a controlled setting. Our study also builds upon early real-world evidence reported by Sambhara et al., which included both treatment-naïve and treatment-experienced eyes, with shorter follow-up and a mean of 3.38 aflibercept 8 mg injections [15]. In their study, treatment decisions were made at the discretion of multiple investigators without a standardized protocol. In contrast, our study applied a consistent T&E approach within a single-centre setting, allowing for more uniform treatment delivery and analysis across an exclusively treatment-refractory cohort.

We have several key findings. Firstly, switching led to a modest but statistically significant extension of treatment intervals, with a reduction in PED height, while maintaining stable BCVA. Secondly, aflibercept 8 mg was well tolerated, with no cases of IOP elevation requiring intervention, and no serious ocular adverse events observed.

At baseline, our cohort represented eyes with a high treatment burden with persistent disease activity despite prior anti-VEGF therapy. Most eyes had already received a substantial number of injections (mean 26.9). More than two thirds (76.3%) of eyes were switched due to persistent disease activity, while less than one third were switched for interval extension or new disease activity. Following the switch, there was an extension of mean injection interval from 7.7 weeks to 8.7 weeks. This was similar to another real-world study on switching to aflibercept 8 mg in previously treated nAMD eyes which gained between 8.8 and 10.7 days [15]. Notably, a higher proportion of eyes (27.1%) in our cohort achieved an interval of ≥10 weeks compared to baseline.

Anatomical improvements were variable. While CST and Cmax remained stable, there was a statistically significant reduction in PED height following treatment switch (from 232.5 to 211.6 µm). PEDs are often considered difficult to treat in nAMD, although there have been previous suggestions that higher dosage of various anti-VEGF agents may lead to a more rapid or improved anatomical response [16]. The qualitative fluid response following the first dose of aflibercept 8 mg was also variable. Approximately two-thirds of eyes showed improvement, with 15% achieving complete resolution of fluid. One-third demonstrated an initial increase in SRF or IRF. Although the proportions of eyes with SRF and IRF were reduced at the final visit compared to baseline, these changes were not statistically significant.

Approximately one-third of eyes (32.2%) gained between 1 and 4 letters, with an additional 13.6% gaining 5 letters or more. Conversely, around 50.8% of eyes experienced some degree of letter loss, while a small proportion (3.4%) demonstrating no change in BCVA. The final mean BCVA was 68.2 letters, compared to 66.0 letters at baseline, with nearly half of all eyes maintaining a BCVA of ≥70 letters. Despite these individual variations, overall BCVA outcomes remained stable following the switch to aflibercept 8 mg, consistent with findings from other real-world studies of anti-VEGF switching in nAMD [12,13,17,18]. The observed degree of letter loss may be due to several factors. Firstly, our cohort had a relatively high baseline BCVA, with over half of eyes (55.9%) already achieving ≥70 letters at baseline, limiting the potential for significant visual gains. Secondly, the cohort had a mean of 26.9 prior anti-VEGF injections, and many eyes had persistent fluid (89.1%) at baseline—reflecting longstanding disease where structural damage already have occurred. This reflects the high prior treatment burden in this cohort, as well as the well-recognized challenge of maintaining VA gains beyond the first year, in real-world settings [19]. The observed lower odds of BCVA loss among patients treated with aflibercept 2 mg compared to faricimab may reflect a ceiling effect; patients already receiving faricimab may have less room for further functional gains.

In our cohort, aflibercept 8 mg demonstrated a favourable short-term safety profile. One patient experienced a documented stroke between their third and fourth injection, administered 15 weeks apart. However, the patient had pre-existing cardiovascular risk factors, including advanced age and atrial fibrillation, making it unclear whether the event was treatment-related or due to the natural history of their underlying disease. We deemed that the patient was clinically safe to proceed with further intravitreal injections, and the patient subsequently tolerated a fourth and fifth injection without further complications. No other adverse events, such as intraocular inflammation, retinal vasculitis, retinal tears, or endophthalmitis were observed. Importantly, although aflibercept 8 mg has a larger injection volume—0.07 mL compared with the standard 0.05 mL used for most anti-VEGF agents—no cases of IOP elevation requiring pressure-lowering treatment or emergency paracentesis were recorded [6,20,21,22]. This is reassuring, given theoretical concerns that larger injection volumes might increase the risk of transient IOP spikes. Our findings are consistent with the PULSAR trial, where the safety profile of aflibercept 8 mg was comparable to aflibercept 2 mg [8]. Nevertheless, ongoing real-world surveillance remains essential as the use of aflibercept 8 mg expands into broader clinical practice.

The strength of this study is that we provide early real-world data on the efficacy, durability and safety of aflibercept 8 mg following a switch from other anti-VEGF agents in eyes with nAMD. There are, however, several limitations. Firstly, this study is retrospective in nature, with a relatively small sample size and short follow-up period. Secondly, we did not subtype the CNVM lesions using fluorescein angiography or indocyanine green angiography as this was not available to us. However, this reflects real-world clinical practice where treatment decisions are often guided by OCT findings. Thirdly, as with all retrospective studies, the potential for selection bias exists. Nevertheless, real-world analyses are essential for early safety and efficacy insights following the introduction of new therapies and can help guide future prospective studies.

## 5. Conclusions

Switching to aflibercept 8 mg in treatment-refractory nAMD eyes in a real-world setting was associated with increased treatment durability, modest anatomical improvements, stable BCVA, and a favourable short-term safety profile. These early outcomes support the use of aflibercept 8 mg in eyes with high treatment burden and persistent disease activity within routine clinical practice. Importantly, no serious ocular adverse events were observed despite the higher injection volume compared to conventional anti-VEGF agents. Further prospective studies with larger sample sizes and longer follow-up are warranted to confirm the long-term efficacy, durability, and safety of aflibercept 8 mg.

## Figures and Tables

**Table 1:** Baseline characteristics.

Patient level (n = 50)		
**Age, years**	Mean (SD)	80.2 (6.3)
**Male sex**	n (%)	28 (56.0)
**Ethnicity, white**	n (%)	50 (100)
Eye level (n = 59)		
**Pseudophakic**	n (%)	28 (46.7)
**BCVA, letters**	Mean (SD)	66 (14.4)
**≥70 letters**	n (%)	33 (55.9)
**Presence of SRF**	n (%)	39 (66.1%)
**Presence of IRF**	n (%)	25 (42.4%)
**Presence of PED**	n (%)	56 (94.9%)
**Presence of SRF or IRF**	n (%)	53 (89.1%)
**CST, µm**	Mean (SD)	367.2 (100.7)
**Cmax, µm**	Mean (SD)	487.3 (146.6)
**PED height, µm**	Mean (SD)	232.5 (162.4)
Previous anti-VEGF treatment		
**Number of prior injections**	Mean (SD)	26.9 (19.0)
**Most recent interval, weeks**	Mean (SD)	7.7 (1.7)
Treatment before switch to aflibercept 8 mg		
**Faricimab**	n (%)	35 (59.3)
**Aflibercept 2 mg**	n (%)	22 (37.3)
**Brolucizumab**	n (%)	2 (3.4)
**Reason for switching**		
**Persistent disease activity**	n (%)	45 (76.3)
**Interval extension**	n (%)	8 (13.6)
**New disease activity**	n (%)	6 (10.2)

^1^ BCVA, best corrected visual acuity; SRF, subretinal fluid; IRF, intraretinal fluid; PED, pigment epithelial detachment; CST, central subfield thickness; Cmax, maximum central retinal thickness.

## Data Availability

Data are available from the corresponding author on reasonable request.
